# Gender Discrimination in the Workplace: Effects on Pregnancy Planning and Childbirth among South Korean Women

**DOI:** 10.3390/ijerph16152672

**Published:** 2019-07-26

**Authors:** Ji-Hye Kim, Sarah Soyeon Oh, Suk Won Bae, Eun-Cheol Park, Sung-In Jang

**Affiliations:** 1 Department of Public Health, Graduate School, Yonsei University, Seoul 03722, Korea; 2 Department of Medicine, Guro Public Health Centre, Seoul 08299, Korea; 3 Institute of Health Services Research, Yonsei University, Seoul 03722, Korea; 4 Institute of Occupational Health, Yonsei University, Seoul 03722, Korea; 5 Department of Preventive Medicine, Yonsei University College of Medicine, Seoul 03722, Korea

**Keywords:** workplace gender discrimination, sexual discrimination, fertility, pregnancy planning

## Abstract

*Introduction*: This study aims to investigate the association between gender discrimination in the workplace and pregnancy planning/childbirth experiences among working women in South Korea. *Methods*: We analyzed data from the Korean Longitudinal Survey of Women and Families (KLoWF) for the years 2007 to 2016. The study population consisted of 7996 working women, between the ages of 19 and 45. Gender discrimination was measured through the 6-item Workplace Gender Discrimination Scale, evaluating discrimination in terms of recruitment, promotions, pay, deployment, training and lay-offs. Multiple logistic regression analysis was employed to measure the association between gender discrimination and the pregnancy planning/childbirth experience. *Results*: Compared to individuals experiencing no discrimination in the workplace, those experiencing low [odds ratio (OR): 0.78, 95% confidence interval (95% CI) 0.61–0.99] or medium (OR: 0.69, 95% CI: 0.54–0.89) levels of discrimination had decreased odds of pregnancy planning. Likewise, individuals scoring low (OR: 0.70, 95% CI 0.54–0.92), medium (OR: 0.68, 95% CI: 0.51–0.92), or high (OR: 0.47, 95% CI: 0.27–0.80) levels of discrimination also had decreased odds of childbirth experience when compared to the no-experience group. When stratified by income, compared to individuals experiencing no discrimination in the workplace, those experiencing gender discrimination had decreased odds of pregnancy planning for low income (low OR: 0.64, 95% CI: 0.45–0.92; medium OR: 0.55, 95% CI: 0.52–0.97; high OR: 0.45, 95% CI: 0.24–0.87), medium income (medium OR: 0.53, 95% CI: 0.37–0.77; high OR: 0.29, 95% CI: 0.14–0.63), and high income groups (low OR: 0.64, 95% CI: 0.49–0.84; medium OR: 0.69, 95% CI: 0.52–0.92). *Conclusions*: The present study finds that gender discrimination in the workplace is associated with decreased odds of pregnancy planning/childbirth experience among working South Korean women. Furthermore, low and medium income groups were especially more likely to be affected by the level of gender discrimination in the workplace when planning pregnancy.

## 1. Introduction

With a period total fertility rate (TFR) of 1.20, South Korea currently has the lowest fertility rate among member countries of the Organization for Economic Cooperation and Development (OECD) [1]. Considering that the total fertility rate required to ensure a broadly stable population, assuming no net migration and unchanged mortality, is 2.10 children per woman, this is a major social problem.

In response, since 2006, the Korean government has adopted three five-year plans that encourage people to marry and have children [2]. While the five-year plans aim to achieve a TFR of 1.50 by 2020, they have had limited success. Amidst the ‘Third Plan for Ageing Society and Population’ program which increased government spending on family and social protection reforms (extension of maternity leave, establishment of parental leave, expansion of public childcare provision etc.), South Korea’s TFR dropped as low as 1.17 (Statistics Korea, 2017). This may be because, while government spending on family and social protection reforms has increased [2], Korea’s expenditure on maternity and parental leave per child born is still one of the lowest among the OECD [3]. Spending on maternity and parental leave has always been low, despite entitlements that cover a relatively long period, and scholars assume that this is because uptake rates are also particularly low compared to other OECD countries [4]. Despite leave entitlements for both men and women, there is evidence that gender stereotypes affect uptake rates, and gender-based stigmas and negative attitudes in the workplace limit both men and women’s uses of leave even when they are entitled to it [5].

Gender discrimination in the Korean labor market is assumed by scholars to be associated with Korea’s low fertility rate [2,6]. Such discrimination occurs when there is a bias in the recruitment, selection and development opportunities among job candidates or workers, who are alike in all respects except their gender [7]. Gender discrimination is associated with multiple health problems and unhealthy behaviors, ranging from stress [8], social anxiety [9] and depression [10], to drinking and cigarette smoking [11]. Verniers and Vala state that certain myths imbuing women with specific abilities for domestic and parental work result in the traditional distribution of gender roles being maintained, and more blatant gender inequalities in the workplace [12]. Worldwide, gendered discrimination in employment results in women’s decisions to postpone or to refuse childbearing [13] from fear that employers have less interest in hiring, promoting and educating working mothers [14]. Many women may also quit their jobs following childbirth to raise their children, and this is likely to affect their pregnancy planning decisions [15]. Widespread fear of discrimination by employers against pregnant women, new mothers and women with small children has been associated with women’s decisions to postpone or refuse childbearing [7,13,16]. For example, in Poland, working women find it difficult to give birth unless there is a babcia (grandmother) who is available and willing to provide childcare [13]. In Japan, highly educated, working women are reluctant to take on the gendered burden of marriage because of the “normalized gendered path of quitting a job on marriage and childbirth” and a “lack of female role models in the workplace” [17]. In Korea, employed women have more decreased odds of giving birth to a second child than do employed women [6], especially if they have a low labor market standing.

However, few studies in Korea have attempted to empirically investigate the association between gender discrimination in the workplace and its effects upon pregnancy planning and childbirth in a nationally representative study of working women. More research is necessary to determine the effects of such discrimination on childbirth among families, as well as how various sociodemographic mechanisms affect this relationship.

In South Korea, even though the attitudes and practices of fatherhood are generally evolving [18,19], discrimination emerges in the male-oriented work culture and gender pay gap, with women’s earnings only amounting to 62% of that of men’s [1]. In one nationally representative study, 58.2% of working women reported to suffer from gender discrimination in terms of income, compared to 5.2% of men, while 79.3% of women reported gender discrimination in terms of promotion opportunities, compared to 3.9% of men [16]. Furthermore, with the average maternal age of first delivery being 31.4 years, fewer women in the 30–34 age bracket (52.9%) are employed than women in the 25–29 age bracket (66.2%) [2]. Over time, the gender wage gap, defined as the difference between male and female median wages, divided by the male median wage), has been closing in South Korea: (South Korea’s wage gap 2000: 41.7%; 2005: 39.6%; 2010: 39.6%; 2015: 36.6%) [20]. However, the gender wage gap has been decreasing for the entire OECD on average, and compared to OECD percentages, South Korea’s gender wage gap is still relatively high (OECD’s wage gap average 2000: 17.7%; 2005: 15.6%; 2010: 14.5%; 2015: 14.2%) [20].

Moreover, the level of gender discrimination in the labor market is consistently changing; influenced by various mechanisms, including gender models inside and outside the family. Factors ranging from the increasing postponement of marriage and parenthood, the rise in marital instability, and the disconnection between marriage and childbearing, all affect the association between workplace discrimination and pregnancy planning among couples [21]. Furthermore, gender roles and stereotypes for males and females vary for each individual, depending on how the concept of gender socialization has been transmitted from parent to child, with regard to housework gender division, and/or attitudes toward the opposite sex [22]. Such factors can result in different responses towards workplace discrimination when it comes to pregnancy planning and childbirth.

Thus, considering South Korea’s ‘lowest-low-level’ fertility decline [23] and widespread gender discrimination in the workplace, the present study investigates the association between gender discrimination in the workplace and pregnancy planning/childbirth experience among working women in South Korea, while controlling for various sociodemographic and individual characteristics.

## 2. Materials and Methods

### 2.1. Study Population and Data

This study was conducted using data from the Korean Longitudinal Survey of Women and Families (KLoWF) for the years from 2007 to 2016. The KLoWF is a longitudinal study conducted by the Korean Women’s Development Institute (KNSO Certificate Number: 33801). A multistage sampling design was used to stratify a nationally representative sample of women from 9,084 households out of all of the urban and rural districts of Korea, excluding Jeju Island. The survey population consists of a total of 10,013 adult women between the ages of 19 and 64 (response rate: 95.8%), who have been tracked every two years since 2007. Surveys are conducted by trained interviewers who conduct face-to-face interviews through a computer-assisted personal interview (CAPI) system.

The KLoWF questionnaire is divided into three sections pertaining to the household, the individual and the individual’s economic activity. In the household section, questions regarding family relationships, household income, housing and consumption, are asked. In the individual section, general questions include those about educational attainment, marital status, pregnancy, childbirth and family planning, and family values. In the economic activity section, economic experience, job search experience, job satisfaction level, discrimination and social insurance questions are asked. Further details of the survey design and methods have been published by the Korean Women’s Development Institute [24].

For the purpose of our study, we identified 7996 subjects who had no missing data for the workplace gender discrimination, and/or pregnancy planning/childbirth experience part of the survey. As with other studies relative to childbirth employing this dataset, our data consisted of women aged between 19–45 years old [25,26]. We also made sure that all of our subjects were working women with occupations that could be categorized into private, public, and/or other (religious, NGO) organizations. Ethical approval was not required, as KLoWF provides secondary, anonymous data that is publicly available for scientific use.

### 2.2. Variables

#### 2.2.1. Pregnancy Planning/Childbirth Experience

The dependent variables in this study were pregnancy planning and childbirth experience. Pregnancy planning was asked in the household section of the survey through the following question: “Do you have plans to have children?” Possible responses included (1) Yes, (2) No. Individuals who selected the ‘yes’ response were categorized into the pregnancy planning group, and individuals who answered ‘no’ were categorized into the non-planning group.

Childbirth experience was found through the Birth ID given to each woman’s household. Under each woman’s Household ID, for each child she gives birth to, the KLoWF assigns a Birth ID. Thus, a woman who has five children will have five Birth IDs under her Household ID. Through the number of Birth IDs each woman has, we were able to calculate whether or not she had an experience of childbirth. Women who had no Birth IDs under her Household ID were categorized into the non-childbirth experience group, while women with one or more Birth IDs were categorized into the childbirth experience group.

#### 2.2.2. Gender Discrimination in the Workplace (Workplace Gender Discrimination Scale)

The Workplace Gender Discrimination Scale is a six-item scale that measures gender discrimination in the workplace through an evaluation of discrimination in the following six areas: Recruitment, promotions, pay, deployment, training and lay-offs. Frequently used as a measure for evaluating workplace discrimination among Korean scholars [26,27], respondents are asked to express their degree of agreement on a 4-point Likert Scale shown (Table 1). In our analysis, the sum of all six questions were added for a total score between 0 (strongly disagree with all six items) and 18 (strongly agree with all six items). Then the respondents were categorized into the following four groups: No gender discrimination in the workplace (when scoring 0 on the scale), low level of discrimination in the workplace (1–6), medium level of discrimination in the workplace (7–12), or high level of discrimination in the workplace (13–18).

#### 2.2.3. Covariates

For this investigation, individual (age, educational attainment, occupation type, self-perceived health), household (income, husband’s employment status, current economic status), and workplace (gender discrimination in the workplace, workplace region) covariates were controlled for in all statistical models.

### 2.3. Statistical Analysis

Chi-square tests were used for all variables to evaluate and compare the general characteristics of the study participants. Multiple logistic regression analysis was employed to analyze the association between gender discrimination in the workplace and the pregnancy planning/childbirth experience. Subgroup analysis was performed to investigate the combined effects of workplace gender discrimination and socioeconomic status on pregnancy planning/childbirth experience outcomes. Odds ratios (ORs) and 95% confidence intervals (CIs) were calculated to compare the prevalence of the pregnancy planning/childbirth experience among study subjects according to the level of discrimination experienced.

## 3. Results

Table 2 presents the general characteristics of the study participants. Results show that 9.2% (*n* = 737) of our study population had plans to become pregnant while 18.3% (*n* = 1463) had an experience of childbirth. The most common level of gender discrimination experienced by subjects was “low” with 43.1% (*n* = 2539) of the study population being in this category. This was closely followed by the “medium” level with 31.8% (*n* = 2539) of subjects.

Table 3 reveals the association between gender discrimination in the workplace and pregnancy planning/childbirth experience. Compared to individuals experiencing no discrimination in the workplace, those experiencing low [odds ratio (OR): 0.79, 95% confidence interval (95% CI) 0.71–1.00] or medium (OR: 0.65, 95% CI: 0.47–0.93) levels of discrimination had decreased odds of pregnancy planning. Likewise, individuals scoring low (OR: 0.80, 95% CI 0.4–0.92), medium (OR: 0.75, 95% CI: 0.48–0.90), or high (OR: 0.57, 95% CI: 0.39–0.80) levels of discrimination had decreased odds of childbirth experience when compared to the no-discrimination group.

**Figure 1:**
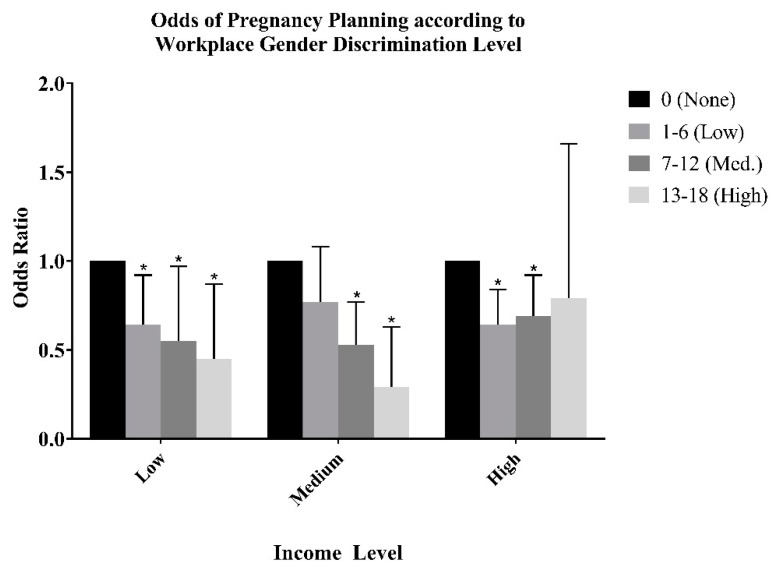
Odds of Pregnancy Planning according to Workplace Gender Discrimination Level, Stratified by Income group.

Figure 1 reveals the results of the subgroup analysis, examining the combined effects of gender discrimination in the workplace and income on the odds of pregnancy planning. When stratified by income, compared to individuals experiencing no discrimination in the workplace, those experiencing gender discrimination had decreased odds of pregnancy planning for low income (low OR: 0.64, 95% CI: 0.45–0.92; medium OR: 0.55, 95% CI: 0.52–0.97; high OR: 0.45, 95% CI: 0.24–0.87), medium income (medium OR: 0.53, 95% CI: 0.37–0.77; high OR: 0.29, 95% CI: 0.14–0.63), and high income groups (low OR: 0.64, 95% CI: 0.49–0.84; medium OR: 0.69, 95% CI: 0.52–0.92). However, while there was an exposure-response relationship between the gender discrimination level and decreased pregnancy planning among the low and medium income groups, for women in the high income group, those suffering from medium levels of gender discrimination had greater odds of pregnancy planning than those suffering from low levels of gender discrimination.

## 4. Discussion

This study examined the association between gender discrimination in the workplace and any pregnancy planning/childbirth experience among working women in South Korea. Three important findings were found. First, there was a statistically significant association between gender discrimination in the workplace and pregnancy planning. 

Second, there was also a strong statistically significant association between gender discrimination in the workplace and childbirth experience. Third, there was an exposure-response relationship between gender discrimination and the decreased odds of pregnancy planning among low and medium income groups. However, this relationship was broken in high income groups, where women suffering from medium levels of gender discrimination in the workplace had greater odds of pregnancy planning than women suffering from low levels of gender discrimination.

The first two findings of our study are both consistent and inconsistent with previous studies worldwide. On the contrary, in a model-based macroeconomic estimate of the cost of gender-based discrimination, Cavalcanti and colleagues found that gender discrimination in general decreases the output per capita by discouraging female labor market participation and therefore, increases fertility [28]. Such findings imply that eliminating gender discrimination in the workplace encourages female labor market participation, and ultimately, results in decreased fertility [28]. Yet it is noteworthy that countries investigated in this investigation included countries like Iran and Saudi Arabia, that have ranked extremely low on the Global Gender Gap Index, created by the World Economic Forum to track a country’s progress of gender equality in health, education, economy and politics [29].

Regarding our investigation’s third finding, to our knowledge, the association between workplace gender discrimination and childbirth planning has not been stratified by income groups in previous studies. However, this finding is important, as it has multiple political implications. As emphasized by Kim and colleagues, the pro-natal policies currently employed by the South Korean government will not be effective unless there is a greater sense of job security among young people [30], especially among individuals in low or medium income groups, rather than high income groups. Social and educational reforms that improve the social status of women and gender equity in general are compulsory, as well as more measures to guarantee the involvement of fathers in childcare and rearing responsibilities [30]. Likewise, while the government has been implementing various policies that provide more favorable working environments for women, a complete elimination of gender discrimination in the workplace is crucial [30]. For such elimination to ensue, the government must provide more childcare facilities for female workers, as well as flexible working hours and short-term leave for family-related purposes among women with young children [30].

There are several limitations to our study. First, our study was conducted using cross-sectional association data, which cannot be used to clarify whether gender discrimination in the workplace precedes the postponement/rejection of pregnancy planning/childbirth, or occurs as a result of certain behaviors by women who postpone/reject pregnancy planning/childbirth. Thus, information regarding gender discrimination that was available in 2007 may have been more difficult to aggregate over time, as well as data regarding other variables. Furthermore, our study subjects may have had various life events over time that brought them to change their opinions, which must be taken into account when interpreting our data results. For example, many women may quit their jobs following childbirth or in the first years of giving birth to raise their children, and this may affect their future pregnancy planning decisions [15].

Second, there may be unrecognized confounding factors, as is true for all observational studies. For example, as aforementioned in our manuscript, multiple mechanisms, including gender models inside and outside the family, may affect the association between workplace gender discrimination and the childbirth experience. Although the extent of these mechanisms could not be evaluated because they were not part of our survey instrument, both micro- (e.g., partnership type, housing and time commitments) and macro-level factors (e.g., secularization, the ideology of responsible parenthood, growing post-materialism, the empowerment of women and changing expectations towards motherhood and parenthood) influence low fertility [31]. Depending on the consistently-changing economic progress, institutional modernization, and developmental state of a country, birth rates can also increase or decrease as direct and indirect costs borne by parents increase or decrease [32]. Some scholars have even investigated the association between the availability and use of reproductive technologie, s like assisted reproductive technologies (ARTs) in South Korea; arguing that the South Korean government’s heavy systematic involvement in regulating and promoting the use of reproductive technologies discourages couples from giving birth [33]. 

Such confounding factors, including a variation of sampling time, may have contributed to the inconsistent association between workplace discrimination and the pregnancy planning/childbirth experience [34].

In addition, the workplace gender discrimination level was categorized into four categories: None, low, medium, and high, because no previous studies have attempted to categorize workplace gender discrimination scores into levels or cut-off points. Internal consistency, as measured with Cronbach’s alpha, was acceptable for the total score of the scale (Cronbach’s alpha = 0.7), however, future studies must investigate the appropriate cut-off scores for categorizing this scale in the same manner, as well as the extent to which these categories are mono-dimensional.

Lastly, all responses to the six-item workplace gender discrimination scale were measured through opinions and attitudes collected from each individual in a survey format. Therefore, all of the figures are based upon subjective perceptions of individual respondents, and reflect their personal experiences. Although such data is meaningful, it is important to take note that one individual’s perceived level of discrimination is likely to be different from that of another individual’s.

Despite these limitations, the current study has strengths in that our results were obtained from nationally representative data on working women. Our results may show a specific aspect of the association between workplace gender discrimination and the pregnancy planning/childbirth experience that is only researchable in South Korea, where the fertility rate is extremely low, gender discrimination is high, and the nation is absent of policies that completely prohibit gender discrimination in the workplace, as is common among other OECD countries like the United States (Fair Labor Standards Act/Pregnancy Discrimination Act etc.).

## 5. Conclusions

In conclusion, the present study investigates an association between workplace gender discrimination and pregnancy planning/childbirth experience in a nationally representative sample of working women in South Korea. Our findings demonstrate that gender discrimination in the workplace, in terms of recruitment, promotions, pay, deployment, training and lay-offs, have an exposure-response relationship with the pregnancy planning and childbirth experience. Future investigations are needed to elucidate the effects that some further factors, such as low socioeconomic status, have on this association. Likewise, cohort or period effects could be measured in similar studies to determine variations in the association between gender discrimination in the workplace and pregnancy planning for women over time.

## Figures and Tables

**Table 1:** Workplace Gender Discrimination Scale.

Questions	Likert Scale			
	Strongly Agree	Agree	Disagree	Strongly Disagree
Assuming the same level of capability/experience among men and women…				
1. Men are recruited more easily than women	(3)	(2)	(1)	(0)
2. Men are promoted more frequently than women	(3)	(2)	(1)	(0)
3. Men are given more pay and benefits than women	(3)	(2)	(1)	(0)
4. Men and women are allocated different jobs	(3)	(2)	(1)	(0)
5. Men are given more opportunities for job development than women	(3)	(2)	(1)	(0)
6. Women are laid-off more than men	(3)	(2)	(1)	(0)

**Table 2:** General Characteristics of Study Observations (2007–2016).

	Pregnancy Planning						*p*-Value	Childbirth Experience				*p*-Value
	Total		Yes		No			Yes		No		
	N	(%)	N	(%)	N	(%)		N	(%)	N	(%)	
**Workplace Gender Discrimination Level**												
No Discrimination (0)	1722	(21.5)	149	(8.7)	1573	(91.4)	0.7266	326	(18.9)	1396	(81.1)	0.245
Low (1–6)	3448	(43.1)	318	(9.2)	3130	(90.8)		615	(17.8)	2833	(82.2)	
Medium (7–12)	2539	(31.8)	245	(9.7)	2294	(90.4)		480	(18.9)	2059	(81.1)	
High (13–18)	287	(3.6)	25	(8.7)	262	(91.3)		42	(14.6)	245	(85.4)	
**Age**												
19–29	1752	(21.9)	400	(22.8)	1352	(77.2)	<0.0001	711	(40.6)	1041	(59.4)	<0.0001
30–39	2384	(29.8)	325	(13.6)	2059	(86.4)		700	(29.4)	1684	(70.6)	
40–45	3860	(48.3)	12	(0.3)	3848	(99.7)		52	(1.4)	3808	(98.7)	
**Educational Attainment**												
≤Middle School	1271	(15.9)	2	(0.2)	1269	(99.8)	<0.0001	2	(0.2)	1269	(99.8)	<0.0001
High School Diploma	4172	(52.2)	367	(8.8)	3805	(91.2)		781	(18.7)	3391	(81.3)	
≥Bachelor’s Degree	2553	(31.9)	368	(14.4)	2185	(85.6)		680	(26.6)	1873	(73.4)	
**Income**												
Low	2606	(32.6)	201	(7.7)	2405	(92.3)	0.0045	440	(16.9)	2166	(83.1)	0.0004
Medium	2460	(30.8)	251	(10.2)	2209	(89.8)		421	(17.1)	2039	(82.9)	
High	2930	(36.6)	285	(9.7)	2645	(90.3)		602	(20.6)	2328	(79.5)	
**Husband’s Employment Status**												
Employed	3514	(43.9)	304	(8.7)	3210	(91.4)	0.1213	648	(18.4)	2866	(81.6)	0.7683
Unemployed	4482	(56.1)	433	(9.7)	4049	(90.3)		815	(18.2)	3667	(81.8)	
**Husband’s Participation in Housework**												
Satisfactory	4657	(58.2)	380	(8.2)	4277	(91.8)	<0.0001	587	(12.6)	4070	(87.4)	0.7242
Dissatisfactory	3339	(41.8)	357	(10.7)	2982	(89.3)		876	(26.2)	2463	(73.8)	
**Current Economic Status**												
High	46	(0.6)	5	(10.9)	41	(89.1)	<0.0001	15	(32.6)	31	(67.4)	<0.0001
Medium-high	962	(12.0)	118	(12.3)	844	(87.7)		212	(22.0)	750	(78.0)	
Medium	4507	(56.4)	487	(10.8)	4020	(89.2)		941	(20.9)	3566	(79.1)	
Medium-low	1957	(24.5)	115	(5.9)	1842	(94.1)		283	(14.5)	1674	(85.5)	
Low	524	(6.6)	12	(2.3)	512	(97.7)		12	(2.3)	512	(97.7)	
**Occupation Type**												
Private	6363	(79.6)	592	(9.3)	5771	(90.7)	0.2676	1142	(18.0)	5221	(82.1)	0.2624
Public	1339	(16.7)	112	(8.4)	1227	(91.6)		261	(19.5)	1078	(80.5)	
Other (Religious, NGO)	294	(3.7)	33	(11.2)	261	(88.8)		60	(20.4)	234	(79.6)	
**Self-perceived Health**												
Good	538	(6.7)	12	(2.2)	526	(97.8)	<0.0001	19	(3.5)	519	(96.5)	<0.0001
Bad	7458	(93.3)	725	(9.7)	6733	(90.3)		1444	(19.4)	6014	(80.6)	
**Workplace Region**												
Metropolitan	4066	(50.9)	433	(10.7)	3633	(89.4)	<0.0001	777	(19.1)	3289	(80.9)	<0.0001
Urban	3930	(49.1)	304	(7.7)	3626	(92.3)		686	(17.5)	3244	(82.5)	
**Total**	7996	(100.0)	737	(9.2)	7259	(90.8)		1463	(18.3)	6533	(81.7)	

**Table 3:** Factors Associated with Pregnancy Planning & Childbirth Experience.

	Pregnancy Planning				Childbirth Experience			
	Odds Ratio	95% CI			Odds Ratio	95% CI		
**Workplace Gender Discrimination Level**								
No Discrimination (0)	1.00	-		-	1.00	-		-
Low (1–6)	0.79	(0.71)	-	(1.00)	0.80	(0.49)	-	(0.92)
Medium (7–12)	0.65	(0.47)	-	(0.93)	0.75	(0.48)	-	(0.90)
High (13–18)	0.59	(0.38)	-	(1.09)	0.57	(0.39)	-	(0.80)
**Age**								
19–29	25.19	(12.77)	-	(49.66)	43.70	(24.65)	-	(77.49)
30–39	13.59	(7.07)	-	(26.13)	19.65	(11.91)	-	(32.42)
40–45	1.00	-		-	1.00	-		-
**Educational Attainment**								
≤Middle School	0.33	(0.08)	-	(1.44)	0.07	(0.01)	-	(0.55)
High School Diploma	0.84	(0.70)	-	(1.01)	0.92	(0.70)	-	(1.20)
≥Bachelor’s Degree	1.00	-		-	1.00	-		-
**Income**								
Low	1.00	-		-	1.00	-		-
Medium	1.15	(0.91)	-	(1.44)	0.76	(0.58)	-	(1.00)
High	0.96	(0.76)	-	(1.21)	0.86	(0.65)	-	(1.14)
**Husband’s Employment Status**								
Employed	1.00	-		-	1.00	-		-
Unemployed	0.14	(0.11)	-	(0.17)	0.20	(0.14)	-	(0.30)
**Husband’s Participation in Housework**								
Satisfactory	1.00	-		-	1.00	-		-
Dissatisfactory	0.99	(0.20)	-	(4.88)	0.48	(0.15)	-	(1.49)
**Current Economic Status**								
High	1.79	(0.51)	-	(6.25)	17.57	(3.83)	-	(80.61)
Medium-high	1.33	(0.68)	-	(2.63)	4.69	(2.00)	-	(11.01)
Medium	1.57	(0.83)	-	(2.98)	5.36	(2.40)	-	(11.97)
Medium-low	1.25	(0.65)	-	(2.43)	5.05	(2.26)	-	(11.30)
Low	1.00	-		-	1.00	-		-
**Occupation Type**								
Private	0.67	(0.41)	-	(1.10)	0.60	(0.31)	-	(1.16)
Public	0.63	(0.37)	-	(1.08)	0.71	(0.35)	-	(1.42)
Other (Religious, NGO)	1.00	-		-	1.00	-		-
**Self-perceived Health**								
Good	1.00	-		-	1.00	-		-
Bad	0.72	(0.36)	-	(1.45)	0.52	(0.23)	-	(1.13)
**Workplace Region**								
Metropolitan	1.03	(0.86)	-	(1.24)	0.85	(0.66)	-	(1.10)
Urban	1.00	-		-	1.00	-		-

## Declarations:

**Ethics Approval and Consent to Participate:** The need for ethics approval and consent is waived for the KLoWF as it is a public dataset with anonymized data that is impossible to associate with the study participant in any way.

**Consent for Publication:** Not Applicable.
